# Discordance Between Patient-Reported Outcomes and Physician-Rated Motor Symptom Severity in Early-to-Middle-Stage Spinocerebellar Ataxia Type 3

**DOI:** 10.1007/s12311-021-01252-9

**Published:** 2021-03-10

**Authors:** Roderick P. P. W. M. Maas, Dennis J. L. G. Schutter, Bart P. C. van de Warrenburg

**Affiliations:** 1 Department of Neurology, Donders Institute for Brain, Cognition, and Behaviour, Radboud University Medical Center, Nijmegen, the Netherlands; 2 Experimental Psychology, Helmholtz Institute, Utrecht University, Utrecht, the Netherlands

**Keywords:** Patient-reported outcome measures, Spinocerebellar ataxia, Quality of life, Fatigue, Depression, Physical activity

## Abstract

Assessment of patient-reported outcome measures (PROMs) in spinocerebellar ataxias (SCAs) could provide valuable insights into self-perceived health status. Although they are considered additional endpoints in future clinical trials, determinants and interactions of different PROMs in early disease stages remain largely unknown. The aims of the present study were to evaluate health-related quality of life, depressive symptoms, fatigue, and physical activity in mildly to moderately affected SCA3 patients and to examine interrelations between these PROMs and objective disease severity indices. Twenty SCA3 patients and twenty healthy controls of comparable age and sex completed the EQ-5D-5L, Patient Health Questionnaire-9, Profile of Mood States, and International Physical Activity Questionnaire. Disease severity was quantified by the Scale for the Assessment and Rating of Ataxia (SARA) and Inventory of Non-Ataxia Signs (INAS). Mildly to moderately affected SCA3 patients reported lower quality of life (*p* = 0.049), more depressive symptoms (*p* = 0.028), and higher levels of fatigue (*p* = 0.001) than healthy controls. The amount of physical activity did not differ between both groups. Linear regression analyses revealed that quality of life was primarily determined by fatigue and not by ataxia severity, while physical activity was independently associated with SARA score and INAS count but not fatigue. Depressive symptoms were related to disease duration and fatigue but not to markers of motor disease progression. Taken together, decreased quality of life, increased levels of fatigue, and a higher number of depressive symptoms do not merely reflect motor impairment in early-to-middle-stage SCA3 patients. The observed discordance between patient-reported and clinician-based outcomes indicates that these measures genuinely evaluate distinct aspects of disease and emphasizes their complementariness in therapeutic trials. By contrast, the volume of self-reported physical activity is not associated with fatigue, reflects both ataxia severity and extracerebellar involvement, and could therefore represent a useful marker of motor impairment in a home setting.

## Introduction

The dominantly inherited spinocerebellar ataxias (SCAs) encompass a clinically and genetically heterogeneous group of neurodegenerative disorders. Contrary to what their terminology might suggest, multifocal nervous system involvement is commonly observed in most types. SCA3 is particularly known for its pleomorphic phenotype that frequently also comprises a number of non-motor symptoms and signs [1]. Indeed, varying combinations of ataxia, spasticity, dystonia, parkinsonism, polyneuropathy, cognitive deficits, sleep disorders, depression, and fatigue progressively interfere with a patient’s daily activities and negatively influence quality of life.

Assessment of patient-reported outcome measures (PROMs) can offer valuable insights into self-perceived health status, which typically reflects complex interactions between physical, emotional, and social factors. Due to their multidimensional and subjective character, PROMs provide clinically relevant information, especially in progressive conditions, that may not be easily captured by routinely applied disease severity scales. Accordingly, they have been recommended as additional outcome measures in future SCA trials [2].

Prior investigations among SCA patients revealed reduced quality of life and frequent co-occurrence of depressive symptoms [3–5]. Ataxia severity, number of extracerebellar signs, and presence of depression independently predicted subjective health status but only explained 30.5% of its variance [4]. This implies a substantial role for other factors not covered in the applied multivariate model, such as fatigue, which is often regarded by patients as one of the most troublesome disease-related complaints [6]. Of note, quality of life and depression scores were not specified per disease stage, and studies thus far mainly involved moderately affected individuals [3–5]. In this light, the absence of differences on quality of life and depression scores between preclinical SCA mutation carriers and healthy controls raises a question about the status of these PROMs during the early symptomatic stages and, consequently, about their suitability as endpoints in upcoming therapeutic trials [7]. Indeed, any intervention will probably be most effective early in the disease course, which makes it especially relevant to investigate the utility of potential outcome measures in these individuals [8]. Furthermore, determinants of subjective health status in mildly affected patients remain largely unknown.

Our cohort of SCA3 patients who participate in a tDCS trial offers the possibility to evaluate various PROMs at early disease stages, as well as their associations with more objective, clinician-based disease severity indices. Here, we sought to (1) examine whether instruments assessing quality of life, depression, fatigue, and physical activity reliably distinguish those individuals from healthy controls and (2) identify factors that might determine quality of life, depressive symptoms, and physical activity.

## Methods

### Study Design and Participants

The present study was a cross-sectional analysis of baseline data from the SCA3-tDCS trial. Within this interventional study, we enrolled 20 mildly to moderately affected carriers of a pathogenic repeat expansion in the *ATXN3* gene and examined the effects of cerebellar transcranial direct current stimulation on a variety of outcome measures [9]. Ataxia severity was graded according to the Scale for the Assessment and Rating of Ataxia (SARA), and “mild to moderate disease” was defined by a score between 3 and 20 at a recent visit. Patients were recruited from our ataxia outpatient clinic and through the Dutch ataxia society. Baseline data were acquired between October 2018 and April 2019. In addition, 20 unrelated individuals of comparable age and sex who did not have a history of neurological or psychiatric disease and did not use centrally acting medication or recreational drugs volunteered to participate as healthy controls. The local medical ethics committee (CMO region Arnhem-Nijmegen) approved the study and written informed consent was obtained from all participants.

### Patient-Reported Outcome Measures

Several questionnaires addressing various PROM domains were administered electronically.

The EQ-5D is a widely used generic health-related quality of life instrument that comprises both a descriptive system and a visual analogue scale. The first part contains five multiple choice questions about mobility, self-care, usual activities, pain/discomfort, and anxiety/depression. Given its higher discriminatory power, which is especially relevant when assessing mildly affected patients, we used the version with five rather than the one with three response options per dimension [10]. Response levels of this EQ-5D-5L are no problems, slight problems, moderate problems, severe problems, and extreme problems. Besides a 5-digit code, the descriptive profile of the EQ-5D-5L can be transformed into a summary index that reflects the societal perspective on a particular health state. Index scores range from less than 0 (in which 0 represents a health state equivalent to death) to 1 (full health) and differ per country according to the preferences of its general population. In the second part of the EQ-5D-5L, respondents quantify their overall health on a scale from 0 (worst imaginable health state) to 100 (best imaginable health state).

The Patient Health Questionnaire-9 (PHQ-9) was selected to screen for depression and determine the severity of symptoms. Each of the instrument’s nine items is rated according to its frequency of occurrence during the last 2 weeks from 0 (not at all) to 3 (nearly every day), yielding a sum score between 0 and 27. Following common practice, we classified depression as none to minimal (0–4), mild (5–9), moderate (10–14), or severe (≥ 15) and defined clinically relevant depression by a PHQ-9 score ≥ 10 [11]. In order to ascertain the relative frequency of specific depressive symptoms, we further dichotomized scores at the individual item level into “no problem” (score 0) or “any problem” (score 1 to 3). Items were separately labelled as “critical problem” if they occurred more than half the days or nearly every day. Lastly, although not included in the PHQ-9, patients were asked if they had visited a psychologist in the previous 3 months or currently took antidepressants.

We administered the shortened 32-item version of the Profile of Mood States (POMS) to examine different affective domains simultaneously, i.e., depression, anger, tension, fatigue, and vigor [12]. Participants rate the extent to which each of the 32 presented adjectives fits their mood state at a scale from 0 (not at all) to 4 (very well). This translates into maximum scores of 32 for depression, 28 for anger, 24 for fatigue and tension, and 20 for vigor. Cutoff values per domain have not been specified for the POMS.

The amount of physical exertion conducted in the last 7 days, both job-related and during leisure time, was measured using parts 1 and 4 of the International Physical Activity Questionnaire (IPAQ) (long form) and expressed in Metabolic Equivalent of Task (MET) minutes a week.[13] To this end, the energy expenditure of the given activity—3.3 for walking, 4.0 for moderate-intensity exercise, and 8.0 for vigorous exercise—was multiplied by the number of minutes per day and number of days per week on which that activity was performed. SCA3 patients’ outcomes were compared with World Health Organization (WHO) recommendations.

### Disease Severity Measures

Ataxia severity and extent of extracerebellar involvement were quantified using the SARA and Inventory of Non-Ataxia Signs (INAS), respectively [14, 15]. INAS count ranges from 0 to 16, in which higher scores indicate more severe extracerebellar pathology. Activities of daily living (ADL) were assessed by part II of the Friedreich Ataxia Rating Scale (FARS) through a structured interview [16]. Disease duration, calculated from self-reported age of onset, complemented the evaluation.

### Statistical Analysis

Data are reported as mean and standard deviation (SD), median and interquartile range (IQR), or frequency and percentage, as appropriate. We used the Shapiro-Wilk test to check the normality assumption. Since almost none of the continuous variables followed a normal distribution in healthy controls, Mann-Whitney U tests were employed to analyze between-group differences. Differences in frequency distributions were evaluated by Fisher’s exact tests.

Next, we explored interrelations between PROMs and disease severity measures. Pearson’s correlation coefficients or Spearman’s rank-order correlation coefficients were used for this purpose, depending on the distribution of data. We separately examined the influence of employment status on EQ-VAS and PHQ-9 scores through univariate linear regression models. A distinction was made between being unemployed versus having a paid job or doing volunteer work.

In order to evaluate whether SCA3 patients’ self-perception of overall health matches the valuation of the Dutch general population, we determined the correlation between EQ-VAS scores and EQ-5D summary index values of their descriptive profile. The latter were derived from a previously reported representative sample (*n* = 2367) using a tobit model on the basis of the composite time trade-off technique [17].

To further explore possible associations between SCA3 subphenotypes and quality of life, depressive symptoms, and fatigue in univariate regression analyses, we combined (1) INAS hyperreflexia, extensor plantar, and spasticity items as pyramidal features; (2) INAS rigidity, chorea, dystonia, myoclonus, and resting tremor items as extrapyramidal features; and (3) INAS areflexia, paresis, muscle atrophy, fasciculations, and sensory symptoms as neuropathic features. In addition, we similarly examined the influence of INAS cognitive impairment on these PROMs.

Finally, we employed ordinary least squares regression analysis with backward selection to identify factors that are independently associated with quality of life, depression, and physical activity. SARA score, INAS count, disease duration, and fatigue were selected as independent variables. Results are presented as unstandardized (*b*) and standardized (*β*) regression coefficients. Statistical analyses were performed in SPSS Statistics Version 25. Test results were considered significant at the 0.05 level (two-sided).

## Results

### Clinical and Demographic Characteristics of Participants

Twenty SCA3 patients (51.9 ± 10.0 years; 12 males) and twenty healthy controls of comparable age and sex (53.2 ± 6.2 years; 12 males) participated in this study. SCA3 mutation carriers had a mean age of onset of 43.9 years (SD = 9.2 years), disease duration of 8.0 years (SD = 5.4 years), repeat length of 67.6 (SD = 3.4), SARA score of 11.9 (SD = 3.9), and INAS count of 5.9 (SD = 1.6). Nine of them had a part-time or full-time job. Among the eleven patients who were unemployed, two did volunteer work.

### Health-Related Quality of Life

EQ-5D-5L data are presented in Table 1. Among the five health dimensions, mildly to moderately affected SCA3 patients more often reported problems with mobility (90% vs. 5%, *p* < 0.001), usual activities (55% vs. 5%, *p* = 0.001), and self-care (30% vs. 0%, *p* = 0.02) compared to healthy controls. Differences in problem frequencies at pain/discomfort (55% vs. 25%) and anxiety/depression dimensions (25% vs. 5%) did not reach significance (*p* > 0.1). Consequently, SCA3 mutation carriers had lower EQ-5D index scores (median 0.82, IQR 0.71–0.88) than controls (median 1.0, IQR 0.92–1.0), as determined using Dutch tariffs (*p* < 0.001) [17]

**Table 1:** EQ-5D ratings of mildly to moderately affected patients with spinocerebellar ataxia type 3 and healthy controls

	SCA3 patients	Healthy controls
Mobility		
No problems	2 (10)	19 (95)
Slight problems	7 (35)	1 (5)
Moderate problems	10 (50)	0 (0)
Severe problems	1 (5)	0 (0)
Unable to walk	0 (0)	0 (0)
Self-care		
No problems	14 (70)	20 (100)
Slight problems	6 (30)	0 (0)
Moderate problems	0 (0)	0 (0)
Severe problems	0 (0)	0 (0)
Unable to wash or dress themselves	0 (0)	0 (0)
Usual activities		
No problems	9 (45)	19 (95)
Slight problems	6 (30)	1 (5)
Moderate problems	4 (20)	0 (0)
Severe problems	1 (5)	0 (0)
Unable to do usual activities	0 (0)	0 (0)
Pain/discomfort		
No pain or discomfort	9 (45)	15 (75)
Slight pain or discomfort	8 (40)	4 (20)
Moderate pain or discomfort	2 (10)	1 (5)
Severe pain or discomfort	1 (5)	0 (0)
Extreme pain or discomfort	0 (0)	0 (0)
Anxiety/depression		
Not anxious or depressed	15 (75)	19 (95)
Slightly anxious or depressed	4 (20)	1 (5)
Moderately anxious or depressed	1 (5)	0 (0)
Severely anxious or depressed	0 (0)	0 (0)
Extremely anxious or depressed	0 (0)	0 (0)
EQ-5D VAS	77.5 (62.0–81.5)	85.0 (78.5–90.0)
EQ-5D index value	0.82 (0.71–0.88)	1.0 (0.92–1.0)

Data are expressed as frequency (percentage) or median (interquartile range)

EQ-VAS ratings in SCA3 patients were lower than in healthy controls (median 77.5, IQR 62.0–81.5 versus median 85.0, IQR 78.5–90.0; *p* = 0.049). Compared with scores from a large representative Dutch population sample (*n* = 2367), self-perceived health status was below the average value by age category and sex in 60% of patients. Lastly, EQ-VAS scores were found to correlate well with index values (*r* = 0.64, *p* = 0.002), indicating that patients’ VAS ratings properly reflect the societal perspective on their descriptive profiles.

### Depressive Symptoms

PHQ-9 results are provided in Table 2. Sum scores were higher in SCA3 mutation carriers than in healthy controls (median 3.0, IQR 1.25–5.75 versus median 1.0, IQR 0.0–3.0; *p* = 0.028). Thirty percent of patients were classified as mildly depressed, 5% as moderately depressed, and none as severely depressed, while 15% of the control group fulfilled the criteria for mild depression. One patient had a clinically relevant depression, as defined by a PHQ-9 score ≥ 10, and took an antidepressant. Five others had visited a psychologist in the previous 3 months.

**Table 2:** PHQ-9 results of mildly to moderately affected patients with spinocerebellar ataxia type 3 and healthy controls

	SCA3 patients	Healthy controls
Any problem		
Little interest or pleasure	8 (40)	3 (15)
Down, depressed, or hopeless	3 (15)	1 (5)
Sleep disturbance	12 (60)	9 (45)
Tired or little energy	16 (80)	11 (55)
Poor appetite or overeating	3 (15)	2 (10)
Feeling bad about themselves	5 (25)	1 (5)
Trouble concentrating	5 (25)	3 (15)
Moving or speaking slowly	5 (25)	0 (0)
Better off dead	0 (0)	1 (5)
Critical problem		
Little interest or pleasure	1 (5)	0 (0)
Down, depressed, or hopeless	0 (0)	0 (0)
Sleep disturbance	0 (0)	4 (20)
Tired or little energy	6 (30)	1 (5)
Poor appetite or overeating	0 (0)	0 (0)
Feeling bad about themselves	0 (0)	0 (0)
Trouble concentrating	2 (10)	0 (0)
Moving or speaking slowly	2 (10)	0 (0)
Better off dead	0 (0)	0 (0)
Sum score	3.0 (1.25–5.75)	1.0 (0.0–3.0)
Clinically relevant depression	1 (5)	0 (0)

“Any problem” at the individual item level was defined as a score different from “not at all,” while “critical problem” required a frequency of occurrence on “more than half the days” or “nearly every day.” Data are expressed as frequency (percentage) or median (interquartile range)

At the item level, feeling tired or having little energy (80%), sleep disturbance (60%), and little interest or pleasure in doing things (40%) constituted the most common problems in SCA3 patients. Tiredness was labeled as critical problem in six individuals (30%). Only problems in “moving or speaking slowly” occurred significantly more frequently in patients compared to controls (*p* = 0.047). None of the patients reported suicidal ideation.

### Mood States

**Fig. 1:**
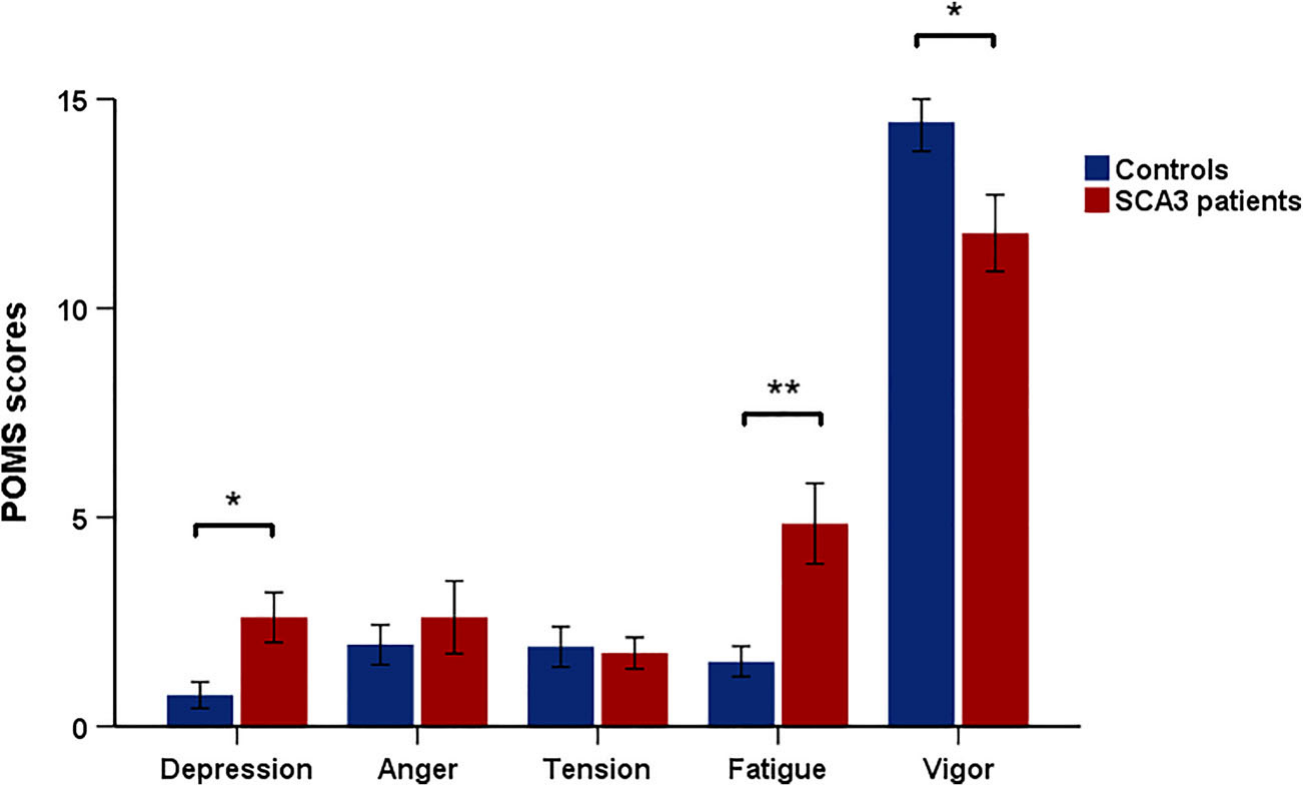
POMS scores of mildly to moderately affected patients with spinocerebellar ataxia type 3 and healthy controls. A single asterisk indicates a *p* value less than 0.05, while a double asterisk indicates a *p* value less than 0.01. Note that maximum scores differ to some extent between the five subscales, as described in the main text. The figure therefore primarily intends to illustrate differences between both groups for each domain and should not be used to directly compare patients’ scores across domains

Data from each of the five POMS subscales are shown in Fig. 1. Compared to healthy controls, SCA3 patients reported higher levels of fatigue (*p* = 0.001) and depressed mood (*p* = 0.012) and lower vigor scores (*p* = 0.038). There were no differences in the domains of anger and tension (*p* > 0.7).

### Physical Activity

**Fig. 2:**
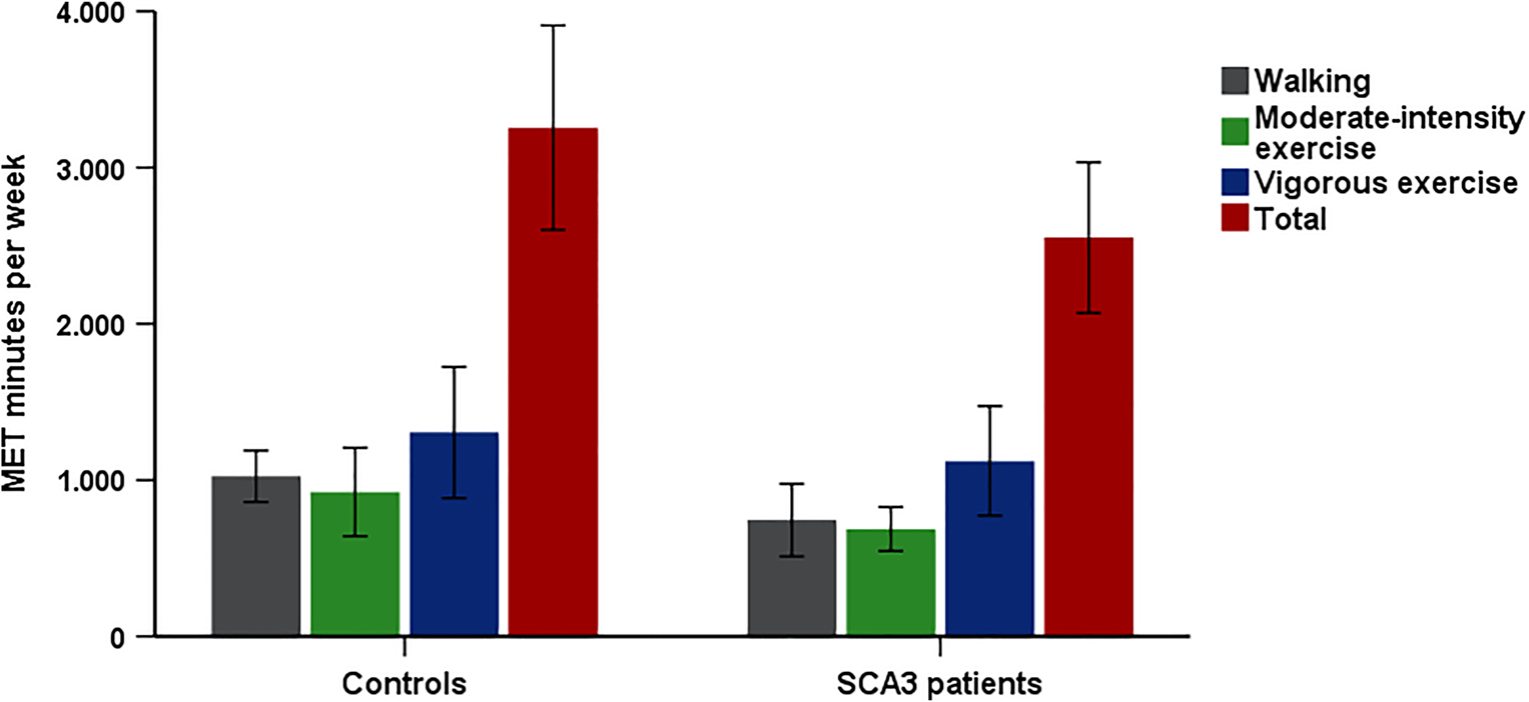
Total number of MET minutes per week and contributions of walking, moderate-intensity exercise, and vigorous exercise in mildly to moderately affected patients with spinocerebellar ataxia type 3 and healthy controls

Figure 2 illustrates the total volume of work-related and leisure-time physical activity that was conducted in the last 7 days by SCA3 patients and healthy controls. The number of minutes spent walking, performing moderate-intensity exercise, and performing vigorous exercise did not differ between both groups (all *p* > 0.05). Twenty percent of SCA3 patients did not meet the WHO recommendations for minimum physical activity, i.e., at least 150 minutes of moderate-intensity physical activity or at least 75 minutes of vigorous-intensity activity throughout the week.

### Associations Between PROMs and Disease Severity Measures

**Fig. 3:**
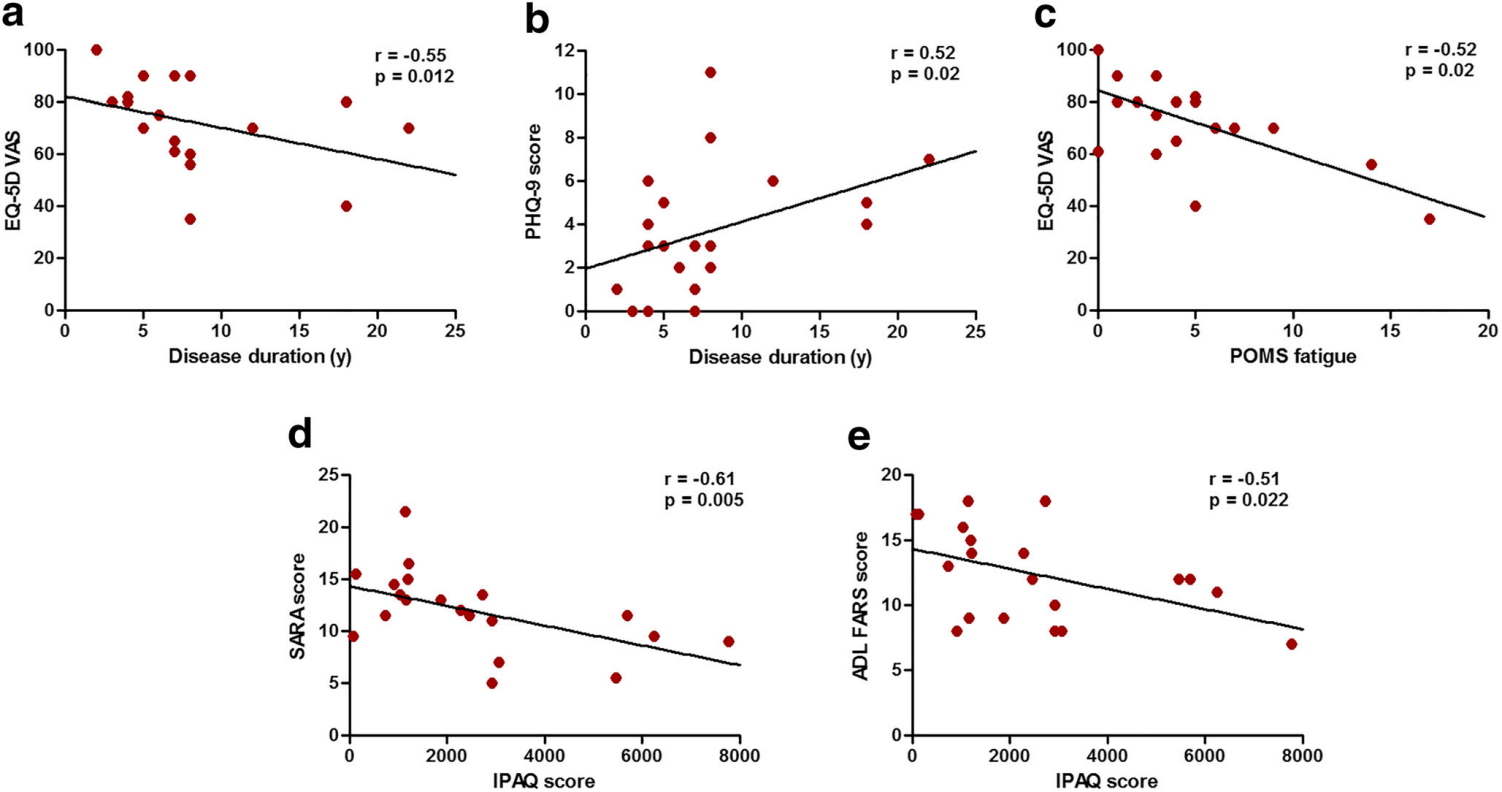
Associations between patient-reported outcome measures and disease severity measures. Shown are correlations between health-related quality of life and disease duration (**a**), between depressive symptoms and disease duration (**b**), between health-related quality of life and fatigue (**c**), between self-reported physical activity and ataxia severity (**d**), and between self-reported physical activity and activities of daily living (**e**)

The interrelations between PROMs and disease severity measures in SCA3 patients can be summarized as follows (Fig. 3). Health-related quality of life and depressive symptoms were associated with disease duration (EQ-VAS: *r* = −0.55, *p* = 0.01; PHQ-9: *r* = 0.52, *p* = 0.02) but not with SARA score (EQ-VAS: *r* = −0.02, *p* = 0.94; PHQ-9: *r* = −0.06, *p* = 0.79), INAS count (EQ-VAS: *r* = −0.40, *p* = 0.08; PHQ-9: *r* = 0.15, *p* = 0.52), or CAG repeat length (EQ-VAS: *r* = 0.02, *p* = 0.94; PHQ-9: *r =* −0.17, *p* = 0.46). This discrepancy might suggest that deterioration of both PROMs in early disease stages occurs independent from motor markers. Lower quality of life was related to higher levels of fatigue (*r* = −0.52, *p* = 0.02). By contrast, the amount of physical activity was inversely correlated with SARA score (*r* = −0.61, *p* = 0.005) and FARS ADL score (*r* = −0.51, *p* = 0.02) but not with fatigue (*r* = 0.00, *p* = 0.99). Lastly, fatigue did not associate with SARA score (*r* = −0.14, *p* = 0.56), INAS count (*r* = −0.07, *p* = 0.76), or CAG repeat length (*r* = 0.09, *p* = 0.72).

Univariate regression analyses showed that unemployed SCA3 patients had lower EQ-VAS scores (*b* = 14.6 [SE = 6.78], *p* = 0.045), higher PHQ-9 scores (*b* = 2.97 [SE 1.14], *p* = 0.018), and higher POMS fatigue scores (*b* = 3.91 [SE = 1.77], *p* = 0.040) than patients who either had a paid job or did volunteer work. SARA ratings, however, did not significantly differ between both groups (*b* = 2.10 [SE = 1.75], *p* = 0.25), indicating that job status in early disease stages is determined by other factors than ataxia severity.

Regarding specific SCA3 subphenotypes, there were no associations between the presence of either pyramidal, extrapyramidal, or neuropathic features and health-related quality of life, depressive symptoms, and fatigue. INAS cognitive impairment, which represents a global subjective assessment of cognitive status, was associated with a higher PHQ-9 score (*b* = 4.67 [SE 1.51], *p* = 0.006) but not with higher levels of fatigue and lower quality of life.

### Predictors of Quality of Life, Physical Activity, and Depressive Symptoms

Multiple linear regression analyses were conducted to identify factors that are independently associated with quality of life, depression, and physical activity. The selected independent variables were SARA score, INAS count, disease duration, and POMS fatigue score. Among these factors, fatigue was found to solely affect health-related quality of life (*b* = −2.45 [SE = 0.69], *β* = −0.65, *p* = 0.002), explaining 41% of its variance. Physical activity, on the other hand, was predicted by both SARA score (*b* = −254 [SE = 101], *β* = −0.46, *p* = 0.022) and INAS count (*b* = −555 [SE = 254], *β* = −0.40, *p* = 0.044), which together accounted for 43% of variance. Finally, fatigue (*b* = 0.50 [SE = 0.09], *β* = 0.74, *p* < 0.001) and disease duration (*b* = 0.17 [SE = 0.07], *β* = 0.32, *p* = 0.029) were independently associated with depressive symptoms, explaining 70% of the variance in PHQ-9 score.

## Discussion

In this study, we compared self-perceived health status, depressive symptoms, fatigue, and physical activity between early-to-middle-stage SCA3 patients and healthy controls and subsequently explored interrelations between these PROMs and more objective, clinician-based disease severity measures. We provide evidence for between-group differences in all PROMs except the volume of self-reported physical activity and describe a dissociation between factors underlying quality of life and physical activity in SCA3 patients. Whereas the former was found to be solely driven by fatigue and not by ataxia severity or degree of extracerebellar involvement, the latter was determined by both cerebellar and extracerebellar motor deficits but not fatigue. The observation that unemployed individuals experienced higher levels of fatigue, yet did not have more severe ataxia than their counterparts with a job, further underscores the functional importance of this common symptom, even in mildly to moderately affected patients.

Quality of life has been previously examined in individuals with spinocerebellar ataxias but not specifically during the early symptomatic stages. EQ-VAS scores from our patients (mean ± SD, 72.7 ± 14.5) were significantly higher than those reported in a European SCA3 cohort with mixed ataxia severities (mean ± SD, 59.1 ± 21.6, difference *p* = 0.001) but lower than scores from preclinical SCA3 mutation carriers (median 85, IQR 78-91) [4, 7]. The finding that EQ-VAS ratings in mildly to moderately affected patients fall between those from preclinical mutation carriers and those across the whole disease spectrum might suggest a gradual decline as a function of ataxia severity. However, quality of life was not associated with SARA score in our study, which is in accordance with data derived from American SCA patients [18]. Instead, fatigue turned out to be its primary determinant during early disease stages, which is an important observation in light of upcoming therapeutic trials. To further ascertain the usefulness of isolated EQ-VAS ratings as potential clinical endpoint, we compared patient and societal perspectives on quality of life by correlating these scores with EQ-5D index scores. The fairly strong correlation between both variables suggests that VAS scores reported by SCA3 patients accurately reflect their descriptive profile’s valuation by the Dutch general population.

Although our results indicate that depressive symptoms are common during the early stages of SCA3, only one patient actually met the criterion for clinically relevant depression. Again, PHQ-9 scores were lower than those from a European (mean ± SD, 6.9 ± 6.2, difference *p* < 0.001) and American SCA3 cohort (mean ± SD, 7.3 ± 5.8, difference *p* < 0.001) with mixed ataxia severities but higher than in preclinical SCA3 mutation carriers (median 2.0, IQR 0.0–3.0) [3, 5, 7]. None of our mildly to moderately affected patients reported suicidal ideation, which is remarkably different from the high rates described in the aforementioned studies (European SCA3 patients: 22.1%; American SCA3 patients: 65.0%) [3, 5]. Despite its high prevalence, it remains largely unknown whether depression in SCA3 arises merely in response to motor impairment or, alternatively, as a result of degeneration of specific neural structures, such as the limbic system, brainstem raphe nuclei, or non-motor basal ganglia-thalamocortical circuits [1, 3, 5, 19, 20]. Depressive symptoms in our study were not related to markers of motor disease progression, such as SARA score and INAS count, arguing against an exclusively reactive origin. Coupled with the independent association between PHQ-9 score and disease duration, this might suggest a role for concomitant degeneration of specific white matter tracts or brain regions implicated in mood regulation, which is in keeping with findings from Lo and colleagues [3]. Interestingly, although there were no specific correlations between depressive symptom severity and pyramidal, extrapyramidal, or neuropathic features, we found an association with the investigator’s overall impression of cognitive impairment, which is consistent with the large EUROSCA study [5].

The data show that fatigue is an early and important symptom in SCA3 patients that negatively impacts quality of life. The precise pathophysiological substrate remains unsettled, but given the widespread nervous system damage that typically occurs, both central and peripheral mechanisms are probably involved [6, 21, 22]. We explored the influence of various extracerebellar signs but observed no association between fatigue and INAS count or between fatigue and pyramidal, extrapyramidal, or neuropathic subphenotypes in particular. The lack of correlation with ataxia severity or disease duration and independent association with depressive symptoms concur with previous investigations [6, 22, 23]. By contrast, Yang and colleagues, while supporting the notion of high levels of fatigue in Chinese SCA3 patients, recently described precisely the opposite pattern of results, i.e., an independent association between fatigue and ataxia severity and a lack of correlation between fatigue and depressive symptoms [24]. We argue that differences in instruments assessing fatigue severity constitute the most likely explanation for this discrepancy. The 14-item fatigue scale (FS-14) that was applied by the Chinese investigators seems to place more emphasis on the somatic component of fatigue (e.g., items like “do you have less strength in your muscles?” and “do you feel weak?”) than the measures used in the other studies. Furthermore, some questions of the FS-14 about mental fatigue may be answered affirmatively by individuals with cerebellar ataxia as a direct consequence of a cerebellar motor deficit and therefore do not necessarily indicate non-motor fatigue. For instance, an affirmative response at the item “do you make slips of the tongue when speaking?” could equally well reflect the presence of dysarthria, which might have introduced a correlation between fatigue and International Cooperative Ataxia Rating Scale score. In keeping with previous work, sleep disturbances were frequently acknowledged by SCA3 patients in the present study as part of the PHQ-9 questionnaire and may negatively affect fatigue severity [6, 23, 24]. However, these were not examined with additional questionnaires or polysomnography, rendering the exact contribution in our population uncertain.

In other neurodegenerative disorders, most notably Parkinson disease, it is well established that physical exercise offers symptomatic motor benefits [25]. Ample evidence also indicates that regular exertion slows nigrostriatal degeneration and thus disease progression [26]. Whether such symptomatic and neuroprotective effects similarly occur in patients with cerebellar ataxia remains less well known. Self-reported assessment of physical activity through standardized instruments, such as IPAQ, could provide valuable information about possible disease-modifying effects of exercise. Yet, to our knowledge, IPAQ has not been previously administered in this population. In contrast to the other three PROMs examined, there were no significant differences in the number of MET minutes per week spent walking, performing moderate-intensity exercise, or conducting vigorous exercise between individuals with SCA3 and healthy controls. Interestingly, SARA score and INAS count were both independent determinants of self-reported physical activity, which implies that IPAQ score could act as composite functional outcome measure reflecting cerebellar and extracerebellar motor impairment.

Our study provides a comprehensive analysis of relevant PROMs and their determinants in the early stages of a rare disease. Limitations are the relatively small sample size, cross-sectional character, and lack of detailed information regarding quality of sleep. As such, the present work has mainly served to generate a variety of hypotheses concerning the interplay between PROMs and disease severity indices. Further multinational collaborative investigations involving larger numbers of patients are required to definitively confirm these relationships.

In conclusion, early-stage SCA3 patients already experience lower health-related quality of life and more depressive symptoms than healthy controls, which appears to be independent of ataxia severity but primarily determined by higher levels of fatigue. Therapeutic strategies that specifically target this disabling complaint should be considered in order to enhance ataxia patients’ well-being. The volume of self-reported physical activity, on the other hand, is not associated with fatigue, reflects both SARA score and INAS count, and could therefore represent a useful marker of motor impairment in a home setting. Lastly, the lack of correlation between depressive symptoms and ataxia severity, extracerebellar involvement in general, and pyramidal, extrapyramidal, and neuropathic manifestations in particular implies that mood disturbances in mildly to moderately affected SCA3 patients do not arise merely in response to motor dysfunction.

## References

[1] Pedroso JL, Franca MC, Braga-Neto P (2013). Nonmotor and extracerebellar features in Machado-Joseph disease: a review. Mov Disord.

[2] Jacobi H, Tezenas du Montcel S, Bauer P, et al. Long-term evolution of patient-reported outcome measures in spinocerebellar ataxias. J Neurol. 2018;265(9):2040–51. 10.1007/s00415-018-8954-0 29959555

[3] Lo RY, Figueroa KP, Pulst SM, Perlman S, Wilmot G, Gomez C, Schmahmann J, Paulson H, Shakkottai VG, Ying S, Zesiewicz T, Bushara K, Geschwind M, Xia G, Yu JT, Lee LE, Ashizawa T, Subramony SH, Kuo SH (2016). Depression and clinical progression in spinocerebellar ataxias. Parkinsonism Relat Disord.

[4] Schmitz-Hubsch T, Coudert M, Giunti P (2010). Self-rated health status in spinocerebellar ataxia--results from a European multicenter study. Mov Disord.

[5] Schmitz-Hubsch T, Coudert M, Tezenas du Montcel S (2011). Depression comorbidity in spinocerebellar ataxia. Mov Disord.

[6] Brusse E, Brusse-Keizer MG, Duivenvoorden HJ, van Swieten JC (2011). Fatigue in spinocerebellar ataxia: patient self-assessment of an early and disabling symptom. Neurology.

[7] Jacobi H, Reetz K, Tezenas du Montcel S, et al. Biological and clinical characteristics of individuals at risk for spinocerebellar ataxia types 1, 2, 3, and 6 in the longitudinal RISCA study: analysis of baseline data. Lancet Neurol. 2013;12(7):650–8. 10.1016/S1474-4422(13)70104-2 23707147

[8] Rubinsztein DC, Orr HT. Diminishing return for mechanistic therapeutics with neurodegenerative disease duration?: There may be a point in the course of a neurodegenerative condition where therapeutics targeting disease-causing mechanisms are futile. Bioessays 2016;38(10):977–80. 10.1002/bies.201600048 PMC5157119 27479863

[9] Maas RPPWM, Toni I, Doorduin J, Klockgether T, Schutter DJLG, van de Warrenburg BPC (2019). Cerebellar transcranial direct current stimulation in spinocerebellar ataxia type 3 (SCA3-tDCS): rationale and protocol of a randomized, double-blind, sham-controlled study. BMC Neurol.

[10] Janssen MF, Bonsel GJ, Luo N. Is EQ-5D-5L Better Than EQ-5D-3L? A Head-to-Head Comparison of Descriptive Systems and Value Sets from Seven Countries. Pharmacoeconomics 2018;36(6):675–97. 10.1007/s40273-018-0623-8 PMC5954015 29470821

[11] Kroenke K, Spitzer RL, Williams JB (2001). The PHQ-9: validity of a brief depression severity measure. J Gen Intern Med.

[12] Wald FDM, Mellenbergh GJ (1990). De verkorte versie van de Nederlandse vertaling van de Profile of Mood States (POMS). Ned Tijdschr Psychol.

[13] Craig CL, Marshall AL, Sjostrom M (2003). International physical activity questionnaire: 12-country reliability and validity. Med Sci Sports Exerc.

[14] Jacobi H, Rakowicz M, Rola R, Fancellu R, Mariotti C, Charles P, et al. Inventory of Non-Ataxia Signs (INAS): validation of a new clinical assessment instrument. Cerebellum 2013;12(3):418–28. 10.1007/s12311-012-0421-3 23090211

[15] Schmitz-Hubsch T, Tezenas du Montcel S, Baliko L, et al. Scale for the assessment and rating of ataxia: development of a new clinical scale. Neurology 2006;66(11):1717–20. 10.1212/01.wnl.0000219042.60538.92 16769946

[16] Subramony SH, May W, Lynch D, Gomez C, Fischbeck K, Hallett M, et al. Measuring Friedreich ataxia: Interrater reliability of a neurologic rating scale. Neurology 2005;64(7):1261–2. 10.1212/01.WNL.0000156802.15466.79 15824358

[17] Versteegh MM, Vermeulen KM, Evers SMAA, de Wit GA, Prenger R, Stolk EA. Dutch Tariff for the Five-Level Version of EQ-5D. Value Health 2016;19(4):343–52. 10.1016/j.jval.2016.01.003 27325326

[18] Ashizawa T, Figueroa KP, Perlman SL, Gomez CM, Wilmot GR, Schmahmann JD, Ying SH, Zesiewicz TA, Paulson HL, Shakkottai VG, Bushara KO, Kuo SH, Geschwind MD, Xia G, Mazzoni P, Krischer JP, Cuthbertson D, Holbert A, Ferguson JH, Pulst SM, Subramony SH (2013). Clinical characteristics of patients with spinocerebellar ataxias 1, 2, 3 and 6 in the US; a prospective observational study. Orphanet J Rare Dis.

[19] Cecchin CR, Pires AP, Rieder CR, Monte TL, Silveira I, Carvalho T, Saraiva-Pereira ML, Sequeiros J, Jardim LB (2007). Depressive symptoms in Machado-Joseph disease (SCA3) patients and their relatives. Community Genet.

[20] Saute JA, da Silva AC, Donis KC, Vedolin L, Saraiva-Pereira ML, Jardim LB. Depressive mood is associated with ataxic and non-ataxic neurological dysfunction in SCA3 patients. Cerebellum 2010;9(4):603–5. 10.1007/s12311-010-0205-6 20734176

[21] Chaudhuri A, Behan PO. Fatigue in neurological disorders. Lancet 2004;363(9413):978–88. 10.1016/S0140-6736(04)15794-2 15043967

[22] Martinez AR, Nunes MB, Faber I, D'Abreu A, Lopes-Cendes I, Franca MC Jr. Fatigue and Its Associated Factors in Spinocerebellar Ataxia Type 3/Machado-Joseph Disease. Cerebellum 2017;16(1):118–21. 10.1007/s12311-016-0775-z 27021342

[23] Yuan X, Ou R, Hou Y, Chen X, Cao B, Hu X, Shang H (2019). Extra-Cerebellar Signs and Non-motor Features in Chinese Patients With Spinocerebellar Ataxia Type 3. Front Neurol.

[24] Yang JS, Xu HL, Chen PP, Sikandar A, Qian MZ, Lin HX, Lin MT, Chen WJ, Wang N, Wu H, Gan SR (2020). Ataxic Severity Is Positively Correlated With Fatigue in Spinocerebellar Ataxia Type 3 Patients. Front Neurol.

[25] Xu X, Fu Z, Le W (2019). Exercise and Parkinson’s disease. Int Rev Neurobiol.

[26] Hou L, Chen W, Liu X, Qiao D, Zhou FM (2017). Exercise-Induced Neuroprotection of the Nigrostriatal Dopamine System in Parkinson's Disease. Front Aging Neurosci.

